# The Birmingham Hospital Saturday Fund

**Published:** 1896-11-14

**Authors:** 


### THE BIRMINGHAM HOSPITAL SATURDAY FUND.

Mr. Smedley is evidently very wroth with The Hospital for having criticised certain schemes of his which were set forth in the *Birmingham Daily Argus* on September 8th, 1896; and a considerable space in the October number of *Forward* is devoted to explanations of his projects and to abuse of us for having drawn attention to them in the way we did. In fact, he goes so far as to say that, "as an example of dishonest journalism, it is probable that it would be difficult to find anything to surpass this action on the part of The Hospital." All this is very amusing, but it does not alter the facts.

We are referred back to an article in the March number of *Forward*, on "A Working Men's Hall," and told that "it is on record as far back as March last that, if this scheme was carried out, it would be carried out by capitalists on public grounds, and in no way was the organisation of the Birmingham Hospital Saturday Fund or its moneys to be employed in the scheme"; and we are further told that, as the scheme was set forth with fulness so far back as last March, "the possibility of any doubt as to the facts is placed beyond question," and we are mightily taken to task for having protested against a misuse of the organisation which was not even contemplated. But many things may happen between March and September. What we criticised had nothing to do with what appeared in *Forward* in March, but was concerned solely with what was stated by the *Argus* in September. According to this, the scheme was definitely one for utilising the Hospital Saturday organisation for other than Hospital Saturday work.

The *Argus* wrote as follows: "Said Mr. Smedley, 'I want to make the Hospital Saturday movement the basis of a big work in the town. The organisation is too strong and too good not to do more work.'" Again, speaking of the same scheme: "It will benefit also by the prestige secured by the twenty-five years' continuous existence and growth of the Hospital Saturday movement. 'Why should we not do something with this great organisation?' said Mr. Smedley with enthusiasm. 'There is no city in the country or in the world where such a splendid organisation exists. The men are ready to work, and trained to work, and there is no limit to its future possibilities.'"

In view of these statements, what becomes of the assertion made in the October number of *Forward*, that "in no way was the organisation of the Birmingham Hospital Saturday Fund . . . to be employed in the scheme." So long as the scheme was merely one for erecting a public building, as described in *Forward*, we said nothing about it; but when the *Argus* described it as one for using the Hospital Saturday organisation for non-hospital purposes then we issued a note of warning. For this we are abused and called all sorts of names by "the monthly journal of the Birmingham Hospital Saturday Fund." We venture to think, however, that the subscribers to that fund will thank us for calling attention to the subject, and that *Forward* would have shown more solicitude for the object it represents if it had disowned the projects of Mr. Smedley for using the Hospital Saturday organisation for the furtherance of his own schemes, as described in the *Argus* of September 8th, instead of spending its energies in abusing those who have always wished well to the fund, and have only criticised proposals to misuse its organisation.

